# Time Resolved Small-Angle X‑ray Scattering of Lyotropic Phase Evolution in Drying Levitated Aqueous Alpha Olefin Sulfonate Droplets

**DOI:** 10.1021/acs.langmuir.6c04771

**Published:** 2026-10-26

**Authors:** Robin Winder, Sepideh Khodaparast, Gabriele Sumanskaite, Andrew Bayly, Ian McRobbie, Michael Rappolt, David Harbottle

**Affiliations:** † School of Chemical and Process Engineering, 4468 University of Leeds, Leeds LS29JT, U.K.; ‡ School of Mechanical Engineering, 4468 University of Leeds, Leeds LS29JT, U.K.; § Innospec Ltd, Oil Sites Road, Ellesmere Port, Cheshire, CH65 4EY, U.K.; ∥ School of Food Science and Nutrition, 4468 University of Leeds, Leeds LS29JT, U.K.

## Abstract

Aqueous surfactant dispersions exhibit rich lyotropic polymorphism as a function of concentration and temperature, yet their structural evolution under the nonequilibrium conditions encountered during drying remains poorly understood. Here, we tracked the phase behavior of acoustically levitated, drying droplets of the commercial anionic surfactant alpha olefin sulfonate (AOS) using in situ time-resolved small-angle X-ray scattering combined with cross-polarized microscopy. Droplets of 20 and 40 wt % AOS were dried at 40 and 60 °C, and each pattern was assigned against recently published equilibrium crystallographic data, allowing coexisting phases to be resolved from characteristic diffraction peaks. Evaporation established a radial concentration gradient that, for the 20 wt % droplet at 60 °C, induced the sequence micellar → hexagonal (within 16 s) → ribbon, with two hexagonal phases coexisting at ∼40 wt % (d_10_ = 4.83 nm) and ∼55 wt % (d_10_ = 4.33 nm) and a ribbon crust at ∼60 wt %. Formation of a dense lamellar crust then impeded water transport from the core, transiently swelling the interior hexagonal phase (d_10_ increasing from 4.80 to 4.95 nm at 20 wt % and 4.72 to 4.90 nm at 40 wt %) before its collapse. A 3D-hexagonal phase formed only at the lowest evaporation rate (20 wt %, 40 °C). In all cases the droplets dried within ∼1000 s into a fully lamellar shell (Bragg ratio 1:2:3) enclosing a hollow core, demonstrating how evaporation rate and initial concentration govern the nonequilibrium phase pathway relevant to surfactant spray-drying.

## Graphical abstract

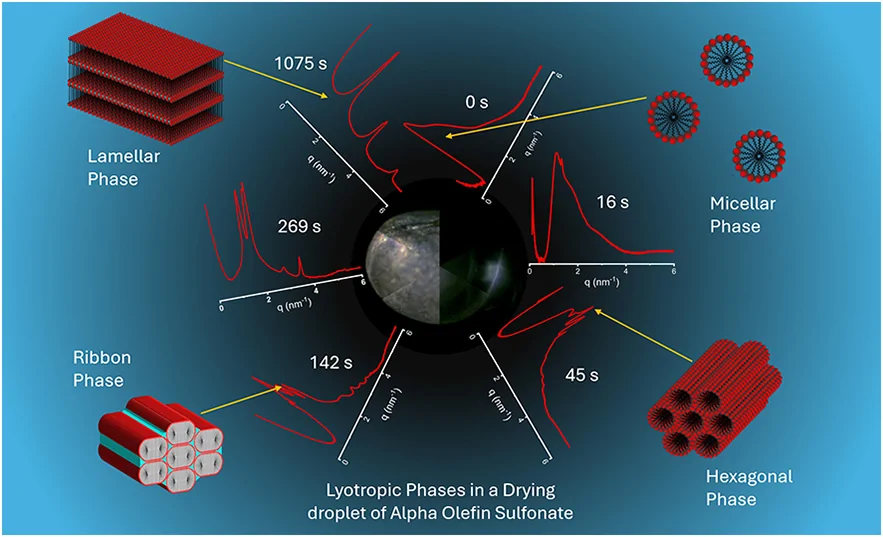

## Introduction

The amphiphilic nature of surfactants, which are comprised of a hydrophilic headgroup and hydrophobic tail group cause them to self-assemble into different aggregates, minimizing contact between the hydrocarbon chains and water while exposing only the polar headgroups to the aqueous phase (hydrophobic effect). These interactions begin once the surfactant reaches its critical micelle concentration, at which point the first ordered structure presents itself. From this point onward, a well-defined series of structures develops, depending on the concentration, temperature and pressure of the system. Micelle formation is followed by the formation of cylindrical micelle aggregates arranged in hexagonal structures and subsequently by bilayers.^1^ Between the hexagonal and lamellar phase, further lyotropic liquid crystal phases may exist including cubic and ribbon phases. The sequence of the phases has been shown to be related to the interfacial curvature and packing constraints, which are governed by the surfactant geometry and the interactions within the system.^2–4^ Studies of these lyotropic surfactant phases typically focus on determining the characteristic phase diagram of a given system as a function of concentration and temperature. This is commonly achieved by examining individual concentrations at different temperatures in near equilibrium conditions.^5–8^ Based on these near-equilibrium phase diagrams, surfactant behavior under more complex environmental conditions can be investigated, enabling the design and optimization of products for specific applications.

The identification and characterization of these phases can be carried out, using techniques such as high definition scanning calorimetry,^5^ nuclear magnetic resonance,^9,10^ cross-polarized microscopy,^11–14^ rheology,^12,15^ and X-ray scattering.^4,16,17^ Combining small-angle X-ray scattering and cross polarized microscopy provides a complementary approach to phase identification. The cross-polarized microscopy reveals changes in birefringence clearly distinguishing isotropic and anisotropic regions. Isotropic regions are typically associated with micellar or cubic phases, whereas anisotropic regions correspond to hexagonal and lamellar phases, each exhibiting characteristic textures and colors.^1^ The scattering patterns obtained from SAXS can then be matched to known phases by the peak position ratios, such as 1:√3:√4:√7 for a hexagonal phase and 1:2:3:4 for a lamellar phase. Furthermore, the peak sharpness and intensity provide information on the degree of long-range order and the relative abundance of the different phases.^18^

Despite the extensive characterization of equilibrium phase behavior in many surfactant systems,^5,7,13^ much less is known about how these structures behave under nonequilibrium conditions. Such conditions arise during both hydration and drying processes, where concentration gradients develop and the system continuously evolves through transient states. Consequently, phase transitions may occur far from equilibrium, often resulting in the coexistence of multiple phases and structures that are not predicted directly from equilibrium phase diagrams. Dissolution solvent penetration studies address such conditions^19–21^ by measuring how quickly and to what depth a liquid solvent diffuses into a solid matrix (like a polymer tablet or film) before the solid dissolves. Further, drying droplets have also been investigated under various environmental conditions, using sessile droplets, where a liquid drop is studied resting on a flat, solid surface,^22–25^ and levitated droplets.^26–28^ The acoustic levitator allows the analysis of the evaporation from droplets in a containerless environment, thus minimizing interactions with surfaces or filaments during the drying process.^29^ This in turn gives insights into phase behavior of drying droplets, which is relevant to spray-drying processes.^30,31^

Water evaporation from surfactant-containing droplets occurs at the air-liquid interface, leading to radial concentration gradients across the droplet.^32,33^ As a result, complex phase behavior is commonly observed, with the coexistence of multiple phases. The evaporation of surfactant-based dispersions has been shown to form a solid crusts^34,35^ on the surface of the droplets, while the interior remain in the liquid and/or gel-like phase. The expected progression is from micellar phases toward increasingly concentrated liquid-crystalline phases, typically including hexagonal and lamellar structures. As these phases exhibit progressively lower water contents, they are expected to be arranged from the droplet interior (water-rich) toward the exterior regions (water-poor). The formation of a dense outer crust influences the subsequent structural evolution of the droplet interior. Here, SAXS reveals its full strength – characteristic peak position ratios enable the identification of coexisting phases, while the corresponding lattice spacings provide information on their hydration state.^36,37^ This forms the framework to understand the drying process of a droplet.

To investigate these drying-induced phase transitions, aqueous dispersions of alpha olefin sulfonate (AOS) were studied. AOS is a commercially relevant anionic surfactant comprising a complex mixture of alkene sulfonates and hydroxyalkanesulfonates with alkyl chain lengths ranging from C_14_ to C_18_. It is widely used in household detergents and personal care products as well as industrial processes such as enhanced oil recovery.^38^ Stable AOS formulations are available in liquid, gel and solid powder forms. As the drying process is the key energy intensive step in manufacturing the solid powder form, understanding the phase evolution within the drying droplet is crucial for identifying the key process parameters that govern the drying behavior. Such insights can ultimately contribute to the optimization of industrial drying processes and consequently, to reducing overall energy consumption.

In this work, we used time-resolved SAXS and cross-polarized microscopy to follow the structural evolution of acoustically levitated AOS droplets throughout drying. Droplets with initial concentrations of 20 and 40 wt % were studied at 40 and 60 °C, enabling us to probe phase transitions originating from both micellar and hexagonal starting states and to isolate the influence of evaporation rate on the subsequent pathway. By assigning each SAXS pattern and cross-polarized image to the equilibrium phases previously established for this system, we resolved the spatial arrangement of coexisting phases within a single droplet and identified the nonequilibrium effects, including transient packing frustration and crust-controlled water transport, that arise when the system is driven far from equilibrium. This approach provides a direct, structurally resolved picture of how a surfactant droplet transforms into a solid particle, a process central to industrial spray-drying yet rarely observed in situ.

## Materials and Methods

### Surfactant Solution

Alpha olefin sulfonate (AOS), a commercial surfactant, was supplied by Innospec Ltd. (UK) at a concentration of 70 wt %, with alkyl chain lengths in the range C_14_–C_16_ and an isomer distribution of 70 wt % 2,3-alkene sulfonate and 30 wt % 3-hydroxyalkanesulfonate. It was diluted with Milli-Q water (Millipore, Burlington, USA; resistivity 18.2 MΩ·cm) to prepare dispersions of 20 and 40 wt %. Each mixture was stirred until homogeneous and then left to equilibrate for 2 weeks before analysis.

### Acoustic Levitator Drying Rig

The setup consists of a TinyLev^39^ acoustic levitator that uses a bespoke heating system that used a Nichrome mesh coil surrounding the droplet to control the drying temperature (Figure 1). This setup has been described in detail in a previous study.^40^ The heating coil was calibrated using a K-type thermocouple (Generic, UK). To calibrate the temperature at the sample position, the current to the heater was adjusted until the desired temperatures of 40 and 60 °C were reached. A 1.8 μL droplet of AOS was then dispensed using a 0.1–2.5 μL Eppendorf Research Plus pipette (Eppendorf, Germany). At room temperature, the droplet was transferred to the central node of the acoustic levitator, inside the heating coil, using a 0.2 mm silver-plated copper wire (Leoni AG, Germany). The quoted drying temperature is the air temperature at the droplet position. It was calibrated by placing a K-type thermocouple of comparable dimensions to the droplet at the acoustic node and increasing the heater current until a stable reading at the target temperature was obtained; the current was then held constant and the droplet introduced. The droplet is surrounded by air, and the flow around it arises from natural convection generated by the heating coil together with acoustic streaming, rather than from any forced flow. The relative humidity at the node is reduced below ambient by the acoustic field and the heater;^31^ at the drying temperatures used here the corresponding vapor pressure deficit is sufficiently large that the residual humidity makes a negligible contribution to the evaporation rate.

**Figure 1:**
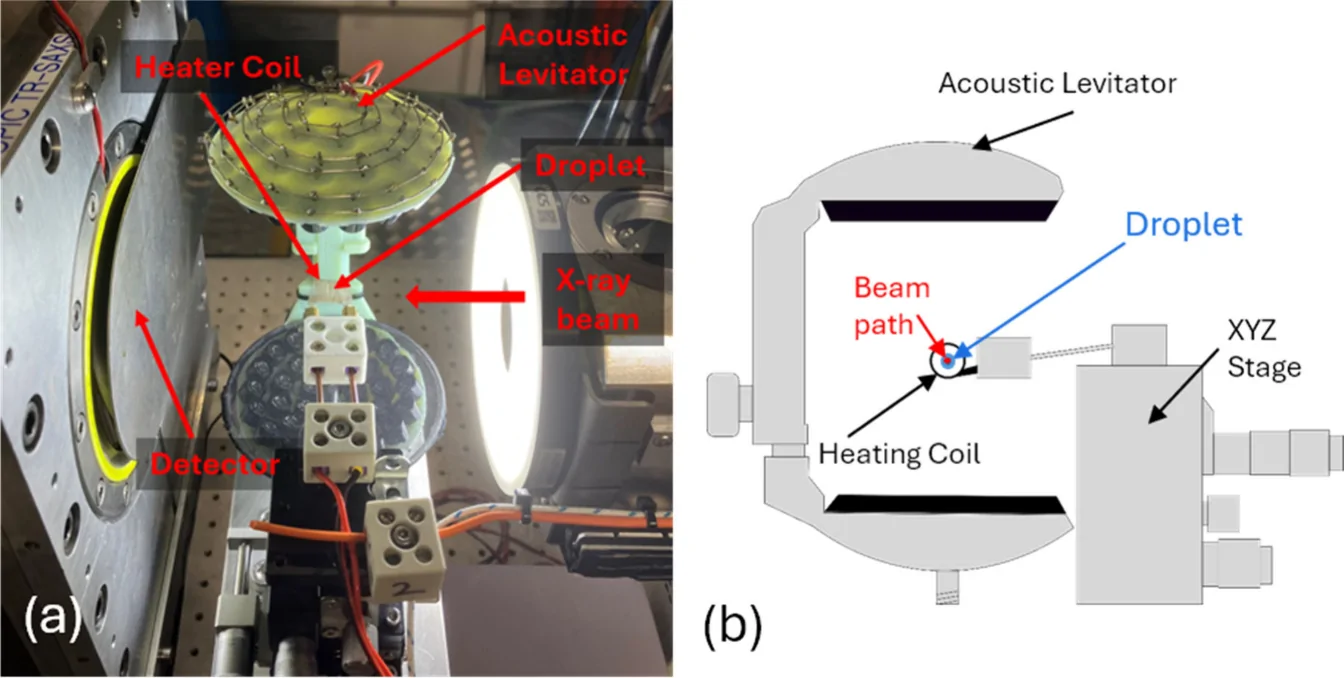
(a) Acoustic levitator drying rig set up on the I22 beamline at the Diamond Light Source. (b) Cross-sectional schematic of the levitator, showing the emitter arrays, the Nichrome heating coil, the droplet at the central node, and the incident X-ray beam path.

### Small-Angle Scattering

SAXS patterns were recorded on the I22 beamline (Diamond Light Source, Harwell, UK) using an X-ray beam of size 200 × 200 μm and a wavelength of 0.0886 nm, corresponding to an energy of 14 keV. The *q* range covered was 0.1 to 9 nm^–1^. Each pattern was recorded with a 0.1 s exposure taken every second throughout the drying process using a Pilatus P3-2 M detector. Droplet oscillation and lateral deviation, arising from convection generated by the heater, continuously refreshed the illuminated sample volume; no evidence of beam-induced damage was observed in any data set. Each condition was measured in repeat experiments, which reproduced both the phase sequence and the transition times (Figure S1).

The peaks obtained at q_hkl_ in reciprocal space were converted to *d*-spacings d_hkl_ using the following equation:

1 $$d=\frac{2\pi }{q},\mathrm{which}\;\mathrm{refers}\;\mathrm{to}\;\mathrm{the}\;\mathrm{Braggs}\;\mathrm{law}\;q=\frac{4\pi }{\lambda }\sin\theta$$

The data were then indexed by assigning Miller indices to each recorded diffraction peak. The lamellar phase is identified by the peak-position ratio 1:2:3. The reflection law is expressed by the relation:

2 $$\frac{1}{d_{h}}=\frac{h}{d}$$

For the 2D hexagonal phase H_1a_ H_1b_ the refection law is given by

3 $$\frac{1}{d_{hk}^{2}}=\frac{4}{3}\frac{h^{2}+k^{2}+hk}{a^{2}}$$

This was verified by checking the ratio of the peak positions, given by 1:√3:√4:√7. For the 2D ribbon phase (M_1_), the Miller indices (hk) were assigned using

4 $$\frac{1}{d_{hk}^{2}}=\frac{h^{2}}{a^{2}}+\frac{k^{2}}{b^{2}}$$

For the 3D hexagonal phase with space group *P*6_3_/*mmc*, the following equation was used to determine the phase assignment:

5 $$\frac{1}{d_{hkl}^{2}}=\frac{4}{3}\frac{h^{2}+{hk+k}^{2}}{a^{2}}+\frac{l^{2}}{c^{2}}$$

The droplet drying rig was placed at the beamline’s sample position. The droplet was aligned with the X-ray beam, and the heater was then switched on to start the drying process, while a series of X-ray patterns wererecorded simultaneously. The resulting 2D data were reduced to 1D SAXS patterns using I22’s standard procedures^41^ within DAWN.^42,43^ The resulting 1D data sets were then compared with bulk measurements of known concentration and temperature at equilibrium to identify the constituent phases of the droplet as it dried.

### Peak Referencing Data

The peak reference set was extracted from our equilibrium phase study^44^ by selecting regions of the phase map in which a single phase is present and plotting all the peak positions accessible to that phase across the concentration and temperature range studied. The only exception is the hexagonal phase, which at the low concentration coexists with a residual micellar contribution (Figure 2); this region was included to capture the full range of hexagonal peak positions, so the hexagonal reference retains a micellar contribution.

**Figure 2:**
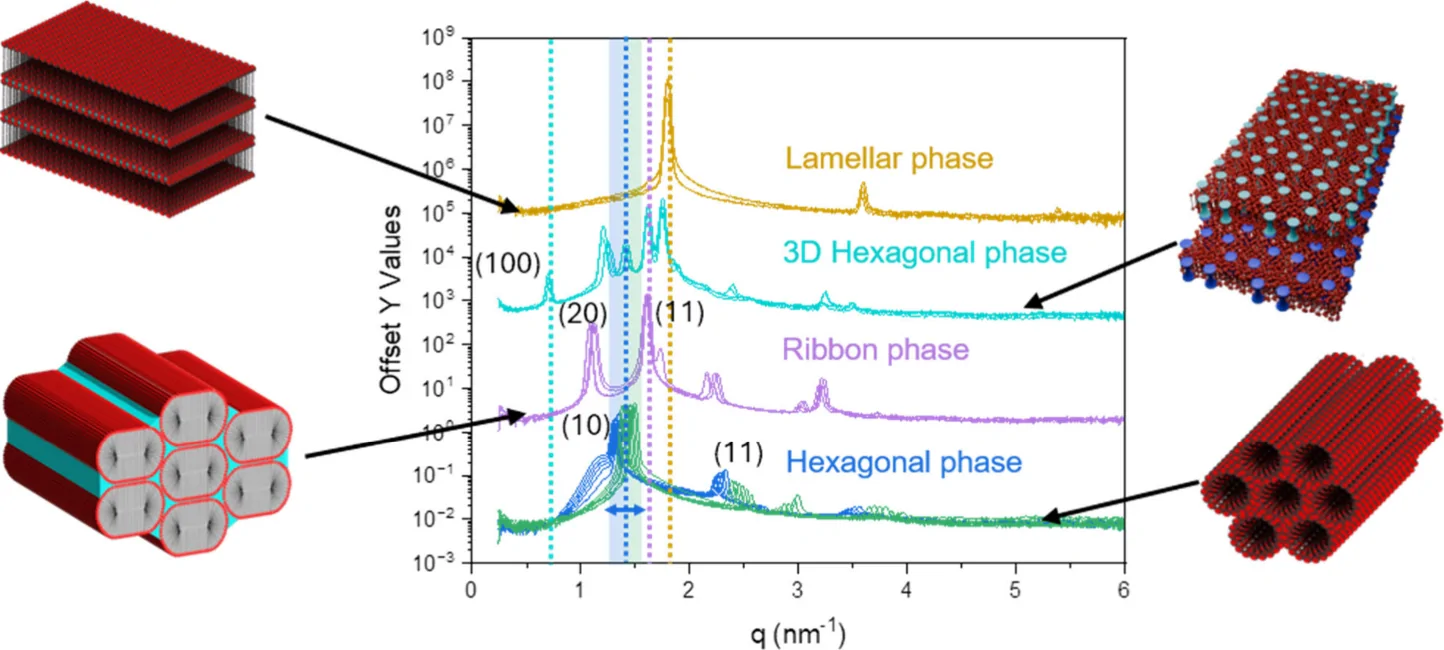
SAXS data and schematics of the four phases formed by alpha-olefin sulfonate in aqueous dispersion (20–40 wt %). The dashed lines highlight the key peaks used to identify individual phases within a mixed system. All reference patterns were taken from single-phase regions of the equilibrium, with the exception of the lamellar pattern, which was taken from a fully dried sample, and the low-concentration hexagonal pattern, which retains a residual micellar contribution. Note that the hexagonal phase spans a wide range of lattice spacings, indicated by the double-headed arrow, depending on concentration and temperature; this phase is color-coded green and blue to distinguish the higher- and lower-concentration variants, respectively.

### Cross-Polarized Imaging

Levitated droplets were imaged using a digital camera (acA1920–150uc, Basler, Germany) fitted with a telecentric lens of 0.8× magnification (TEC-M08110MP, Computar, UK) and a high-contrast linear polarizing filter (PG120-S30, MidOpt, USA). The droplet was illuminated with an LED light source with a built-in polarizer (SLZ_W1, Dino-Lite, Taiwan). Images were captured at 1 fps throughout the drying process. The same optical arrangement was used with the polarizing filters removed to follow the change in droplet size. The principal axes of the droplets were determined from the recorded images using a custom MATLAB script, and the droplet volume was calculated by treating the droplet as an oblate spheroid (Figure S4).

## Results and Discussion

To interpret the nonequilibrium phase evolution occurring during droplet drying, the observed SAXS patterns were first compared with the equilibrium phase behavior of AOS established in our recent phase-map study,^44^ which spans a broad range of concentration (2–70 wt % AOS) and temperature (25–80 °C). The equilibrium phases thus serve as reference states against which the structural evolution of the drying droplets can be evaluated. In the following discussions, all lyotropic AOS phases are described by their space group, lattice spacing, and the structural organization of the surfactant and water domains. Each phase additionally exhibits at least one characteristic diffraction peak that can serve as a unique phase identifier. The four principal phase-identifying peaks are indicated in Figure 2 by dashed vertical lines.

### Equilibrium Phase Identification (Figure 2)

The defining peak for the hexagonal phase is the (10) reflection, which spans a wide range of *q* values (1.26–1.49 nm^–1^) owing to variations in hydration and temperature. This peak lies at a similar position to the (200) reflection of the 3D hexagonal phase (*P*6_3_/*mmc*); however, the defining peak of that phase is the (100) reflection at *q* = 0.7 nm^–1^. Because no other phase exhibits a peak at this intermediate *q* value, the 3D hexagonal phase can always be distinguished from phases with otherwise overlapping peaks. The defining peak for the ribbon phase is the (11) reflection, which remains confined to a narrow region centered around *q* = 1.6 nm^–1^. The only other phase sharing this peak is the 3D hexagonal phase, which, as noted above, is readily ruled out. The final phase is the lamellar phase, identified by a peak at 1.79 nm^–1^. Because the accessible positions of each diagnostic peak are given in unique scattering regimes, individual coexisting phases can be identified unambiguously within a single droplet pattern throughout drying. In the following sections this reference set is applied to all the time-resolved SAXS patterns recorded during drying, allowing the coexisting phases and their hydration states to be tracked.

### Phase Evolution in a 20 wt % Droplet Dried at 60 °C

Throughout the drying process the phase transitions are driven by the change in local composition as water is removed, rather than by temperature: over this concentration range the phase boundaries shift only weakly with temperature,^44^ and evaporative cooling is expected to hold the droplet below the set drying temperature for most of the drying period. Temperature acts indirectly, by setting the evaporation rate and hence the time available for each structure to form, and the spatial arrangement of the phases follows the radial concentration gradient established by surface evaporation.

An initial concentration of 20 wt % was selected because it lies well within the micellar region, allowing the full drying pathway from a micellar solution through each intermediate mesophase to be followed, while retaining sufficient surfactant for the droplet to remain stably levitated throughout drying. A drying temperature of 60 °C provides an enhanced evaporative driving force while keeping the phase evolution slow enough, adequate for the one second sampling rate. Drying at 80 °C was also examined and gave a closely similar phase sequence; the 60 °C data set is presented here as it resolves the phase sequence in clearer manner.

At the start of the drying process (t = 0 s), the cross-polarized microscopy image showed no sign of birefringence, demonstrating that the initial phase is isotropic (Figure 3a). This isotropic phase is a normal micellar phase, characterized in the SAXS data by a single broad peak at low *q*. Modeling the pattern with a core-shell ellipsoid form factor combined with a Hayter-Penfold structure factor for charged particles returns an ellipsoidal micelle with a hydrophobic core of semiaxes 2.11 and 3.17 nm and a polar shell of thickness 0.54 nm, giving overall micelle semiaxes of 2.65 and 3.71 nm and an aspect ratio of approximately 1.4. The core equatorial semiaxis is comparable to the length of an extended C14–C16 alkyl chain, as expected for a normal micelle. A spherical core-shel model could not reproduce the measured scattering profile. Full fitting parameters are given in Table S1 and the fit is shown in Figure S2 of the Supporting Information. In addition, at a concentration of 20 wt %, a broad peak was observed at 1.08 nm^–1^ (Figure 3b). Checking this peak position against our reference set for the possible phases (Figure 2) allows all of them to be ruled out, and we therefore attribute the peak to a structure-factor contribution arising from the onset of micelle aggregation. This structural interaction in the present concentration regime can be attributed to electrostatic repulsion, which arises from partial dissociation of the sodium ions and effectively leaves the micelles charged.^45–49^ The same behavior has been demonstrated for other surfactants such as SDS,^50,51^ where increasing micelle concentration was shown to reduce the intermolecular distances. This aggregation step correlates with a structural reorganization of the micelles, primarily involving their elongation.^52^ The first phase observed during drying is therefore micellar.

**Figure 3:**
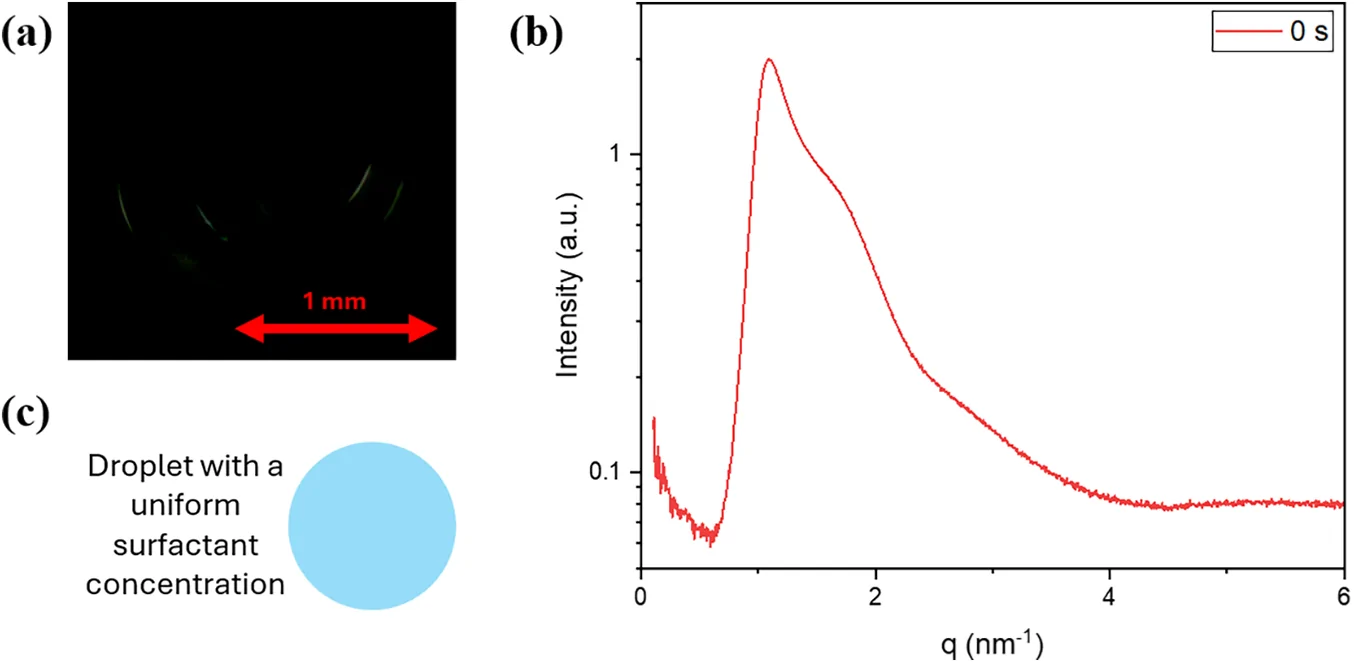
(a) Cross-polarized image of a 20 wt % AOS droplet at the onset of drying at 60 °C, showing the phase to be isotropic. (b) SAXS pattern exhibiting a single broad peak, indicating the presence of micelles together with a structure factor arising from interparticle interactions. (c) Schematic showing a droplet with an initially uniform surfactant concentration at the start of the drying process.

At t = 16 s and a drying temperature of 60 °C, the first signs of an anisotropic phase were detected by cross-polarized imaging, appearing as a faint white cloud-like structure (Figure 4a). These birefringent features were located at the droplet surface rather than in its interior. High-speed cross-polarized imaging (100 fps, Figure S3) resolves two birefringent bands separated by an optically transparent gap; the nearer band remains in focus and follows the curvature of the droplet surface, while the further band is diffuse, as expected from the shallow depth of field of the imaging system. As the droplet oscillates, the two bands converge and the transmitted intensity rises above that of the separated bands, consistent with the near and far walls of a birefringent shell moving into optical superposition so that their retardations combine. Note, a birefringent region confined to the droplet interior would not produce this behavior. The observation was reproduced across repeat experiments. These images also show that the birefringent layer is initially patchy, nucleating in discrete regions before becoming continuous; the gradient responsible for the phase sequence is radial, from the droplet surface inward, and we do not observe a sustained difference in phase behavior between lateral positions on the surface. Simultaneously, the SAXS pattern revealed a sharp peak at 1.3 nm^–1^ and a very weak peak at 2.27 nm^–1^. Their peak-position ratio of 1:√3 justifies assignment to the normal hexagonal phase. The corresponding unit cell spacing of 5.58 nm (eq 3 applied to *q*_10_ = 1.3 nm^–1^) falls within the range established in the equilibrium experiments^44^ for a hexagonal phase at 40 wt % and 60 °C (Figure 2). The underlying micellar structure has changed very little at this stage. This initial hexagonal phase marks the beginning of the crust that forms on the droplet surface. The assignment of the droplet interior to a micellar phase rests on the SAXS data rather than on the optical images: the broad low-*q* peak and its associated interaction peak (structure factor contribution) at 1.08 nm^–1^ remain present at this time point. We note that the curvature and depth of the droplet can cause position dependent changes in polarization through reflection and refraction at the surface, together with specular highlights, so a continuous birefringent layer can appear nonuniform in a single frame; apparent variations in birefringence across the image should not be read as variations in structure. This initial hexagonal phase marks the beginning of the crust that forms on the droplet surface.

**Figure 4:**
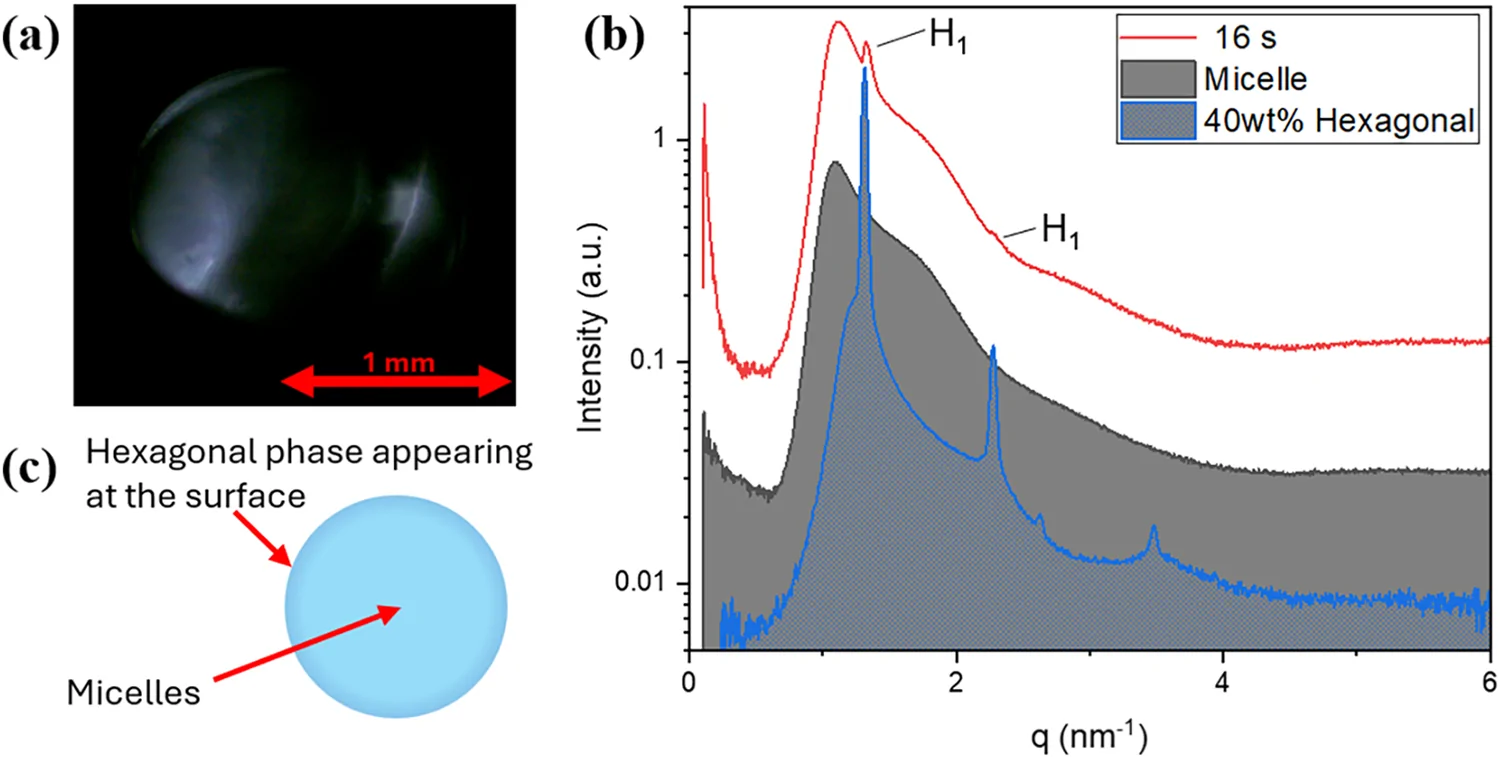
(a) Cross-polarized image of the droplet after 16 s of drying (initial concentration 20 wt %, drying temperature 60 °C); the image now shows some anisotropy, indicated by the cloudy white texture. (b) The red line shows the SAXS pattern of the drying droplet. The underlying components (shaded gray) represent the phases present within the droplet at this time, namely the coexistence of the micellar phase (black line) with traces of a hexagonal phase (H_1_). The latter is identified by Bragg peaks at 1.3 nm^–1^ and 2.27 nm^–1^, with a peak-position ratio of 1:√3. For comparison, the SAXS pattern at 40 wt % (blue line) displays the first four diffraction peaks of the hexagonal phase. (c) Schematic of the droplet showing the onset of the concentration change to a hexagonal phase at the droplet surface.

At t = 45 s the surface structure began to appear, showing positive birefringence with a broader range of diffracted colors and textures which are more consistent with a ribbon phase than the open textures of the hexagonal phase^44^ (Figure 5a). The SAXS data now displayed a series of sharp peaks. The peak at 1.3 nm^–1^ (blue in Figure 5b) remains consistent with the 40 wt % hexagonal phase (H_1a_) observed earlier, but it is now joined by a second peak at 1.45 nm^–1^ (green in Figure 5b), corresponding to a hexagonal phase with a unit cellspacing of 5.00 nm and a local concentration of 55 wt % (H_1b_). Both peaks overlap broadly, which we take as evidence of a continuous concentration gradient between the two rather than a coexistence of two discrete phases; the same profile can be reconstructed from the equilibrium data by superimposing the peak maxima across the intervening concentration range (Figure 2). Because evaporation occurs at the surface, the most concentrated material lies outermost, thus in radial direction the ribbon phase is placed at the surface, followed by H_1b_ and H_1a_ phase being situated further inward (Figure 5c). The weak peak at 2.27 nm^–1^ is the (11) reflection, which is characteristic of the hexagonal phase. In addition, the peak recorded at 2.9 nm^–1^ corresponds to the (20) reflection (√4 Bragg peak) of a hexagonal phase at 55 wt %. We note that the (20) reflection is too weak at lower concentrations and is therefore not initially visible (Figure 2 and Figure 5b). Finally, the ribbon phase (M_1_) was also observed, indicated by peaks at 1.61 nm^–1^ and 1.76 nm^–1^. The (20) peak of this phase is difficult to separate from those of the micellar and hexagonal phases, but a unique peak at 3.24 nm^–1^ allows us to confirm the M_1_ phase (Figure 2). At this point the broad peak of the micellar phase is still superimposed on all of the lyotropic mesophases. The formation of a thin crust, visible in the cross-polarized images, supports the interpretation of a water concentration gradient within the droplet, decreasing from the interior outward, with the ribbon phase forming the crust; under equilibrium conditions this phase exists at concentrations above 55 wt % at 60 °C.^44^ Dissolution studies support this interpretation:^19,53^ as concentrated lamellar phases are slowly dissolved, different nonlamellar phases appear in reverse order, displayed in cross-polarized microscopy as distinct regions in the sequence lamellar, cubic, hexagonal and micellar.

**Figure 5:**
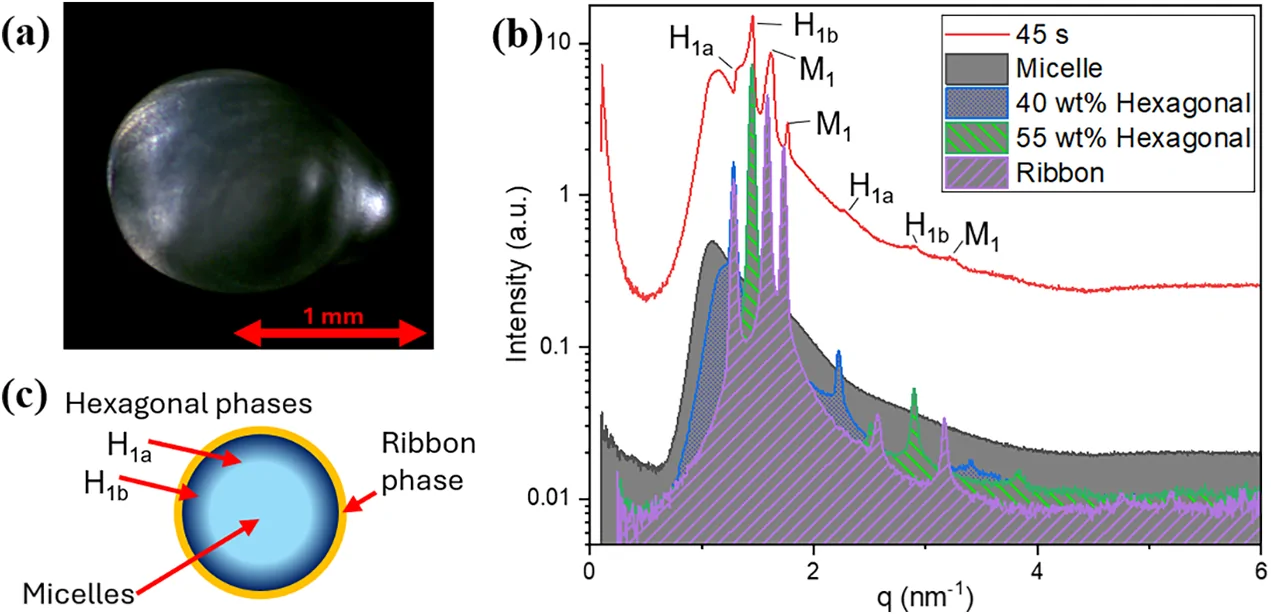
(a) Cross-polarized image of a droplet with an initial concentration of 20 wt %, taken after 45 s of drying at 60 °C. The emerging solid crust displays a range of colors, indicating a higher surfactant concentration at the droplet surface. (b) SAXS data at 45 s of drying (red line). Key diffraction peaks are assigned to an underlying micellar phase coexisting with 40 wt % (H_1a_) and 55 wt % (H_1b_) hexagonal phases and a ribbon phase (M_1_). (c) Schematic of the droplet showing the positions of these phases along a concentration gradient, with the driest phase in the outermost region.

At t = 142 s the cross-polarized image shows a rough crust with considerable texture and color (Figure 6a), demonstrating the thickening of the drying surface. The SAXS data display a significant loss of the broad peak previously seen at 1.1 nm^–1^; that is, the micellar phase has practically disappeared (Figure 6b). Instead, a sharp (20) reflection at 1.20 nm^–1^ of a ribbon phase (M_1_) was now seen, which is clearly distinct from the (10) reflection of the hexagonal phase at 40 wt %, in agreement with near-equilibrium data.^44^ We note that the higher-order diffraction peaks of both coexisting phases are also observed at this stage, owing to their larger crystal sizes and relatively lower lattice disorder. We conclude that the drier ribbon phase forms the crust, with a concentration of 60 wt %, while the interior of the droplet is occupied by the hexagonal phase over a concentration range of 40–55 wt %. It is also worth noting that the diffuse scattering of the micellar phase has tailed off at this point, indicating that the interior concentration has risen and is approaching a purely hexagonal phase.

**Figure 6:**
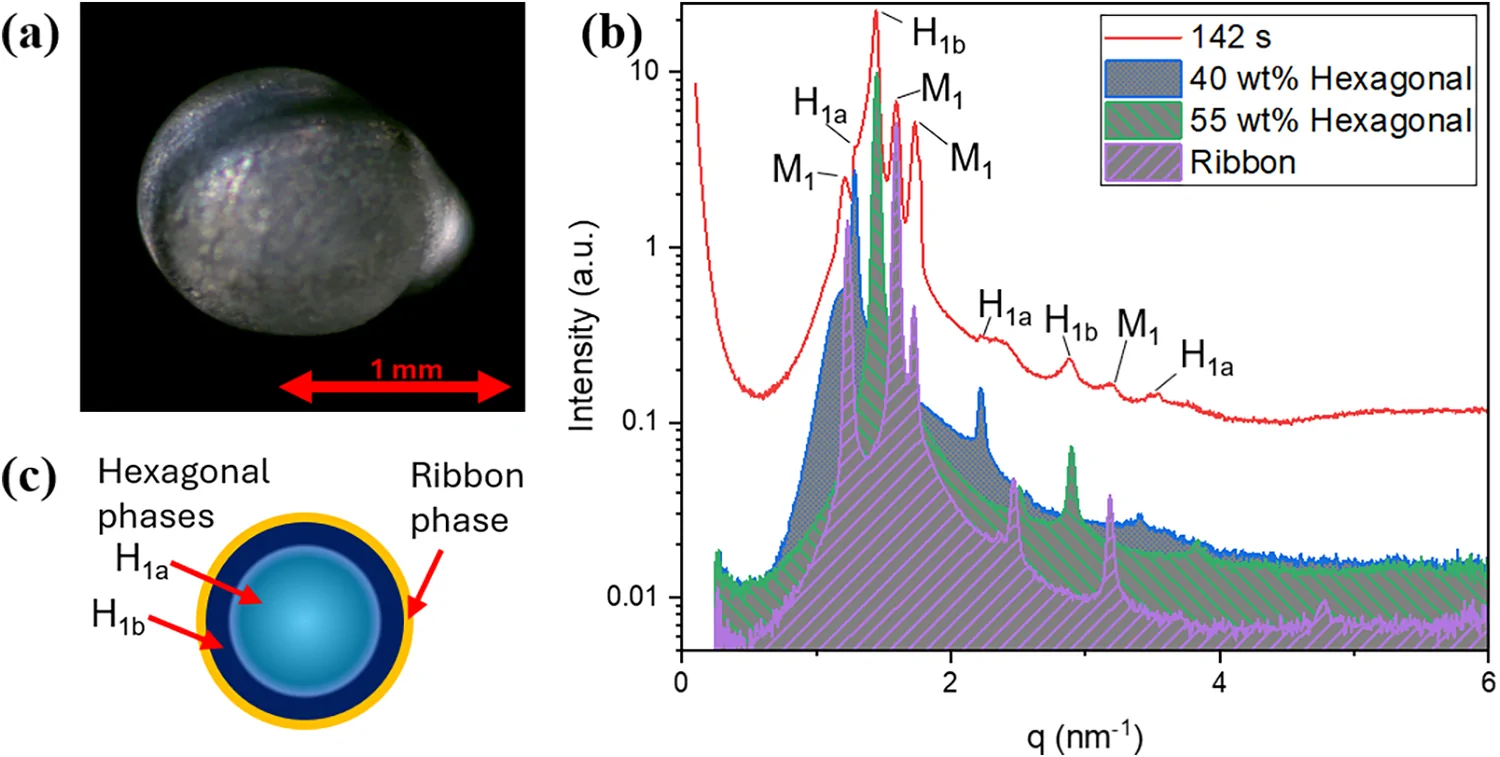
(a) Cross-polarized image of a droplet with an initial concentration of 20 wt %, taken after 142 s of drying at 60 °C. The crust now shows a crumpled texture and a complex series of colors. (b) SAXS data at 142 s of drying (red line). The underlying micellar phase appears to have disappeared, leaving the hexagonal phases (H_1a_, H_1b_) and the ribbon phase (M_1_). (c) Schematic showing the positions of the different phases within the droplet.

At t = 269 s the cross-polarized image begins to show signs of surface denting (Figure 7a). The overall droplet size has changed very little (Figure S4), far less than would be expected if the droplet dried as a fully filled structure. This indicates that a rigid shell forms at the surface early in drying; continued loss of internal water then hollows the droplet and buckles the shell inward, rather than shrinking it uniformly. The peaks in the SAXS data are now straightforward to interpret. A lamellar phase is identified by the strong peak at 1.78 nm^–1^ and its second-order peak at 3.55 nm^–1^, with a peak-position ratio of 1:2. This lamellar phase coexists with a hexagonal phase, identified by its first-order peak at *q*_10_ = 1.42 nm^–1^ and the observation of the √4 peak at *q*_20_ = 2.85 nm^–1^. Although the (11) and (21) reflections are too weak to be recorded in the droplet, direct comparison with equilibrium data^44^ (yellow line in Figure 2) allows us to exclude the coexistence of a second lamellar phase; we further note that the corresponding *d*-spacing of 4.42 nm would be unrealistically large. Consulting the time-resolved contour plot (Figure 10a) confirms the hexagonal-phase assignment: after the 260 s mark, an increase in its lattice spacing was observed. This swelling behavior is not possible for a lamellar phase, which displays a monotonic decrease in lattice spacing on drying.^54^ Taking a closer look at the progression of the first-order peak of the hexagonal phase (yellow arrow, Figure 10a), one observes a broadening and a steady decay in intensity during this stage of drying. Finally, we can exclude the possibility that this peak arises from a micellar phase, since its characteristic broad peak at *q* = 1 nm^–1^ had already vanished at t = 142 s.

**Figure 7:**
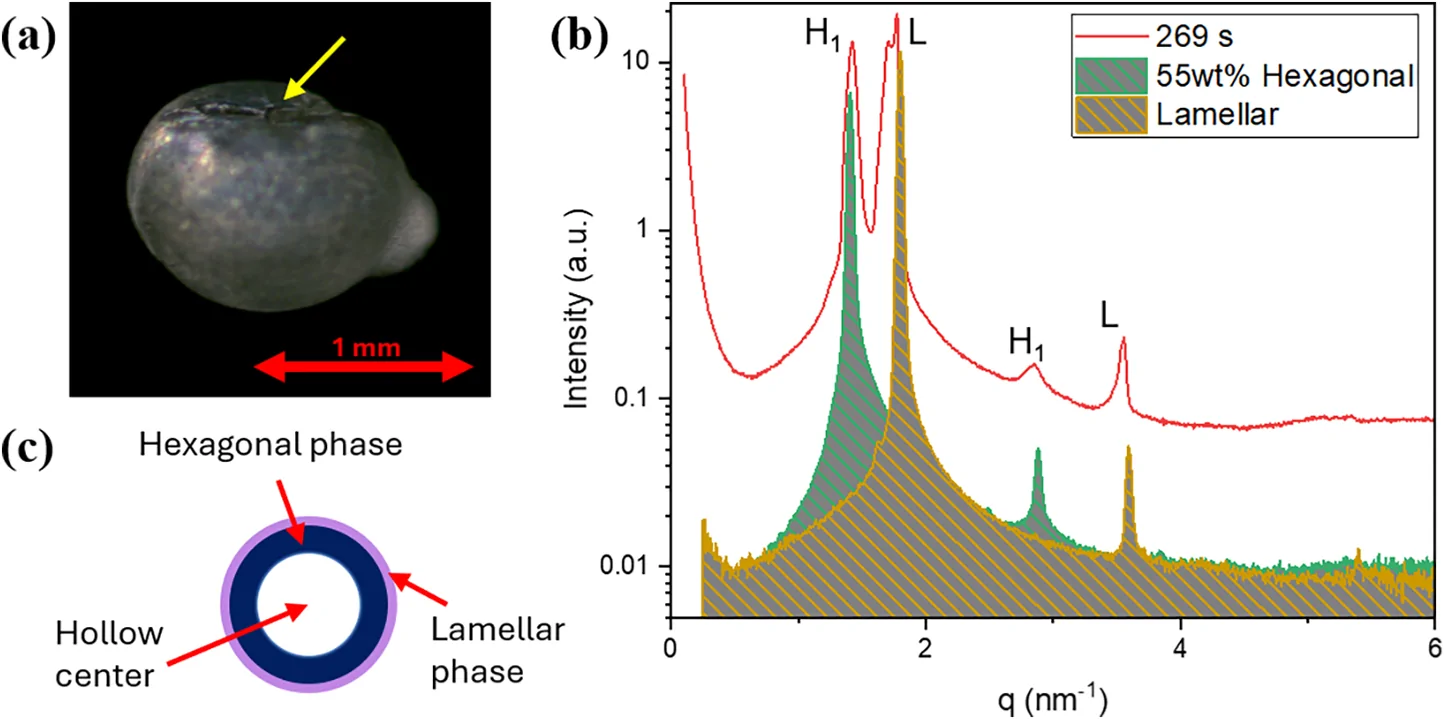
(a) Cross-polarized image of a droplet with an initial concentration of 20 wt %, taken after 269 s of drying at 60 °C. The image shows a cracked, crumpled texture at the top of the droplet (yellow arrow), indicating that a hollow center has formed. (b) SAXS data at 269 s of drying (red line). The reduced number of peaks shows that the crust has reached a lamellar (L) state, while the interior remains wet and in a hexagonal state (H_1_). (c) Schematic showing the hollow center, with a lamellar layer at the droplet exterior and a hexagonal interior.

Most likely, the formation of the dense lamellar phase at the crust has altered the drying dynamics of the droplet. Water transport within the system now relies on defects in the lamellar phase, which hamper the diffusion of water.^55–57^ The internal hexagonal phase depends on long-range order to maintain its 2D structure but, as previously reported, a hexagonal phase can lose its stability under confinement,^58^ which in our case is demonstrated by the broadening of the first-order peak. Finally, the system is unable to remain intact owing to the loss of surfactant, evolving into a single lamellar phase.

At t = 1075 s the cross-polarized image shows little change in size (Figure 8a and Figure S4) but appears darker than previously (a side-by-side comparison is provided in Figure S5), as the dense lamellar crust transmits less light. The SAXS data show only one series of peaks, with a ratio of 1:2:3, denoting a lamellar phase (Figure 8b). The comparative equilibrium SAXS data, recorded on a fully dried sample, show narrower Bragg peaks and display no shoulders. The slight shoulders on the low-*q* side of the droplet reflections indicates a difference in the hydration of the lamellar phase between the interior and exterior of the shell, even after 1075 s of drying, and suggest that the sample was not completely dry when the experiment was terminated. Broader diffraction peaks indicate that less lamellae are given in the dried droplet when compared to equilibrium SAXS data. Microscopy examination of the droplets after drying confirmed that the interior of the droplet was hollow (Figure 9a) and that the crust had formed a layered structure (Figure 9b).

**Figure 8:**
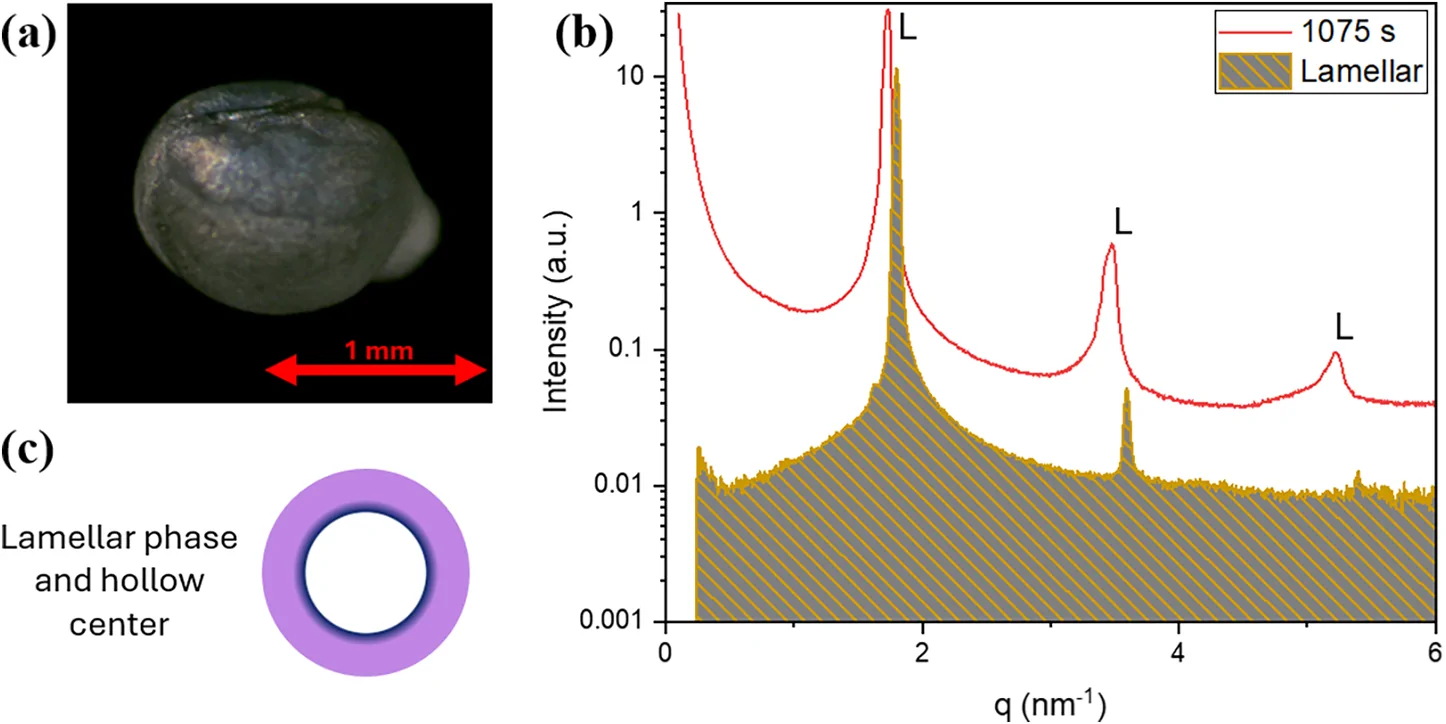
(a) Cross-polarized image of a droplet with an initial concentration of 20 wt %, taken after 1075 s of drying at 60 °C. The image shows a cracked, crumpled texture; note that the volume of the droplet has barely changed, indicating that it is hollow (Figure S4 of the Supporting Information). (b) SAXS data at 1075 s of drying (red line). Only one set of peaks now remains, with the characteristic Bragg ratio of 1:2:3, showing that the droplet has converted entirely to a lamellar state at the end of drying. (c) Schematic showing the dried droplet and its hollow center; the slight shoulders on the peaks are represented by the color change toward the center.

**Figure 9:**
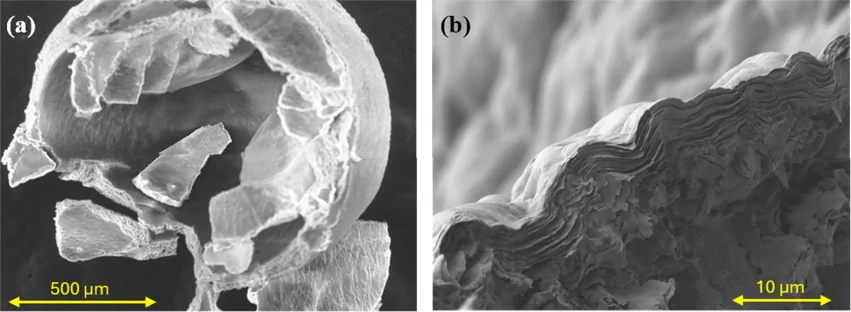
(a) SEM image showing the hollow interior of the droplet. (b) SEM image of a cross-section of the droplet crust.

The layered crust revealed in cross-section (Figure 9b) supports this interpretation: once a continuous lamellar shell is established, water can leave the droplet only through defects in the lamellar structure, and the transport resistance of the shell, rather than the external evaporation rate, becomes the factor controlling the subsequent evolution of the interior. The transient swelling of the interior hexagonal phase is a consequence of this change in the rate-limiting step.

In addition to the 20 wt % sample dried at 60 °C, discussed in detail above (Figures 3–8 and Figure 10a), two further drying processes are shown for comparison: a 40 wt % sample studied at 60 °C (Figure 10b) and a 20 wt % sample at 40 °C (Figure 10c). Changing the drying temperature and the surfactant concentration revealed different phase behavior in the droplets. The initial phase formation relies on an increase in concentration as water evaporates at the surface, driving the hexagonal-to-ribbon and finally the lamellar phase that forms the crust of the droplet (Figure 10a, b); note the absence of the micellar phase in the 40 wt % droplet. Comparing the two initial concentrations, the 40 wt % droplet begins beyond the micellar region of the equilibrium phase map and therefore enters the drying sequence directly at the hexagonal stage; thereafter it follows the same progression as the 20 wt % droplet, through the ribbon phase to a fully lamellar shell. The initial concentration therefore sets the entry point for the phase sequence rather than altering the sequence itself. The principal difference is that the 40 wt % droplet exhibits the transient swelling of the hexagonal phase without the accompanying loss of long-range order seen at 20 wt %, consistent with the shorter period spent in the hexagonal region before the lamellar crust forms. The only exception to this phase sequence was the 20 wt % droplet dried at 40 °C, which showed the 3D hexagonal phase, marked by the red arrow and the overlaid SAXS pattern (Figure 10c). The absence of the 3D hexagonal phase at 60 °C is kinetic rather than thermodynamic in origin. The equilibrium phase map^35^ shows that this phase is accessible at 60 °C at concentrations above 60 wt %, and the drying droplet passes through this composition range; nonetheless the phase does not appear, and the system transforms directly to the lamellar phase. At 40 °C the lower evaporation rate leaves sufficient time for the hexagonally ordered water pockets, a structure of high symmetry, to assemble before the surface water is removed. The fast evaporation rate at 60 °C, rather than temperature acting on phase stability, is therefore the determining factor for suppressing the formation of the 3D hexagonal phase in this case.

**Figure 10:**
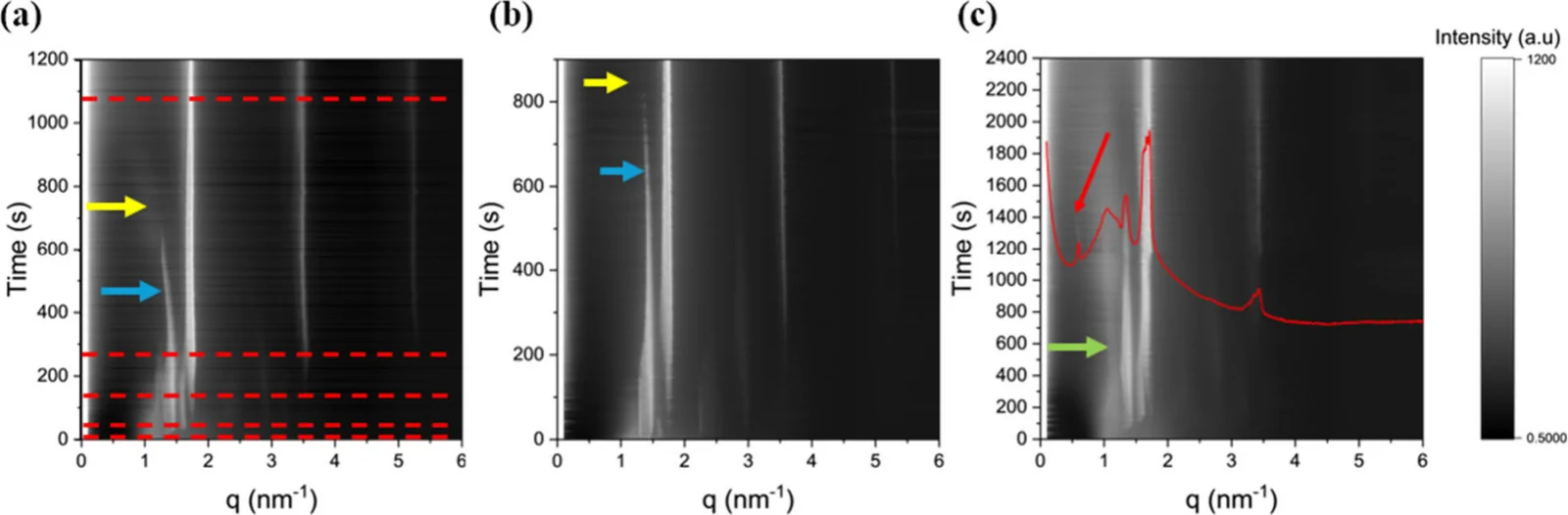
(a) Contour plot of the SAXS data for a 20 wt % droplet dried at 60 °C, corresponding to Figures 3–8 (red dotted lines mark the sampled data). The blue arrow indicates the (10) reflection of the hexagonal phase, and the yellow arrow indicates the breakdown of the hexagonal phase. (b) Contour plot of a 40 wt % droplet dried at 60 °C. Note that no disordered hexagonal phase (yellow arrow) is present after the ordered hexagonal-phase region (blue arrow). (c) Contour plot of the SAXS data for a 20 wt % droplet dried at 40 °C. The overlaid SAXS pattern (red) is assigned to the 3D hexagonal phase by its characteristic peak at low *q* (red arrow). The trend of the micellar phase in the interior of the droplet is indicated by a green arrow.

### Crust Formation and Hindered Water Diffusion

The influence of water diffusion is examined as the drying temperature or surfactant concentration is varied. When the 20 wt % sample is dried at 60 °C, the hexagonal phase formed promptly at t = 8 s and persisted up to t = 708 s (Figure 10a). This phase is short-lived, and its initial hydration is not conserved; the lattice spacing increased from d_10_ = 4.80 to 4.95 nm. When the surfactant concentration is increased to 40 wt % at 60 °C, the hexagonal phase forms even more rapidly, with similar swelling behavior: d_10_ increases from 4.72 to 4.90 nm (Figure 10b). When the 20 wt % sample is dried at 40 °C, the hexagonal phase begins to form only at t = 41 s and persists up to t = 1774 s, but its hydration remains practically constant, with a lattice spacing d_10_ of 4.65 nm throughout its existence. In this case, a micellar phase is also observed to be continuously present throughout the drying process (Figure 10c, green arrow). The hexagonal phase is the only phase in this system that coexists with the micellar phase, both in the reference set (Figure 2) and in the full equilibrium characterization, so the profile in this region is a micellar-hexagonal coexistence rather than a contribution from any other phase. It is worth noting the highestd_10_ value for a hexagonal phase, 4.99 nm, was observed at a concentration of 38 wt % at ambient temperature in the equilibrium studies.^44^

Under equilibrium conditions, an increase in surfactant concentration leads to a decrease in headgroup hydration and tighter surfactant packing, reflected in a reduction of the lattice spacing;^44^ however, there is an exception to this rule under nonequilibrium conditions. When water evaporation is hindered by the formation of the lamellar crust, the hexagonal phase swelled transiently before the hexagonal-to-lamellar transition is complete. This increase in lattice spacing is accompanied by a decrease in intensity and a broadening of the (10) reflection for the 20 wt % dispersions (Figure 10a, c). The hexagonal lyotropic crystals therefore become smaller at this point, comprising shorter and fewer rods, which reduces the attractive van der Waals forces. Importantly, although the water content continues to decrease, albeit at a slower diffusion rate, the ratio of water to surfactant transiently increases. The loss of crystalline order and the reduction in van der Waals forces provide a straightforward explanation for this short-lived swelling of the normal hexagonal phase. The same behavior was reported for a different surfactant blend during the H_1_-to-lamellar crystalline phase transition.^59^ For the lowest concentration, dried at 40 °C, we additionally observed that the structure-factor peak of the micelles at *q* = 1 nm^–1^ never disappeared (Figure 10c, green arrow), demonstrating that the interior of the droplet is at its wettest under these conditions.

In summary: (i) a lower heating temperature (and hence a lower evaporation rate) combined with a higher initial water concentration is essential for the longevity of the micellar phase; (ii) anomalous swelling of the hexagonal phase is apparent only at higher evaporation rates, when the lamellar crust concurrently forms a water barrier; and (iii) the formation of the 3D hexagonal phase requires a lower driving force (lower temperature), because the arrangement of water pockets adopts a complex geometry of high symmetry.

### Relation to Industrial Drying Conditions

The conditions used here differ in several respects from those of an industrial spray dryer. The levitated droplets are approximately 1 mm in diameter, whereas spray-dried droplets are typically 100–500 μm; drying here proceeds over ∼1000 s, against residence times of less than a minute; and the droplet is surrounded by quiescent heated air rather than a hot, dry coflowing gas. These conditions were selected to give the droplet stability and guarantee an appropriate scattering to noise signal required to resolve the phase sequence unambiguously, and the time scale should be understood as a consequence of the enlarged droplet rather than as representative of process duration. The governing physics, however, is the competition between the rate of evaporation at the surface and the rate at which water can diffuse from the interior. Large droplets drying slowly may occupy a similar region of that parameter space to a small droplet drying rapidly. The behavior reported here, ordering that begins at the surface, the formation of a shell, the conversion of that shell into a transport barrier, and the consequent decoupling of the droplet interior from the external drying conditions, follows from that competition rather than from the absolute droplet size or drying time. The kinetic suppression of the 3D hexagonal phase at the higher evaporation rate demonstrates directly that the phase pathway, and therefore the microstructure of the final particle, is set by drying rate and not by composition alone. Extending these measurements toward process conditions will require smaller droplets, higher gas temperatures and controlled gas flow, together with faster detectors to resolve a phase sequence compressed into seconds; the phase assignments and the mechanistic framework established here provide the reference against which such measurements can be interpreted.

## Conclusion

Using combined SAXS and cross-polarized microscopy, we have resolved the complex lyotropic phase behavior of AOS droplets drying at different initial concentrations and temperatures. Five phases: micellar; hexagonal; ribbon; 3D-hexagonal and lamellar, were identified and shown to coexist at successive stages of drying. Evaporation at the droplet surface first drove the expected sequence from micellar through hexagonal to ribbon phases, establishing a lamellar crust at the droplet boundary. Once this dense crust had formed, water transport became dependent on defects within the lamellar phase. Under these confined conditions the interior hexagonal phase lost its long-range order and swelled transiently during its decomposition, a behavior observed for both 20 wt % droplets. The 20 wt % droplet dried at 40 °C additionally developed a 3D-hexagonal phase, previously reported only under controlled equilibrium conditions,^44^ which formed exclusively at this lower evaporation rate, whereas the 40 wt % droplet dried at 60 °C showed hexagonal swelling without the associated disordering. In every case, the droplets dried into a fully lamellar shell enclosing a hollow core.

By interpreting the in situ SAXS patterns against equilibrium reference data, we were able to assign coexisting phases unambiguously and map them to specific regions within the drying droplet. This work demonstrates that the coupling of cross-polarized microscopy and time-resolved SAXS provides a powerful route to characterizing nonequilibrium phase evolution in a drying surfactant droplet, and it offers new insight into how the developing architecture of the droplet governs its internal structural transformations; insight directly relevant to the optimization of energy-intensive surfactant drying processes.

## Supplementary Material



## Notes

Time-resolved SAXS contour plots of repeat droplet-drying experiments (Figure S1); fitting parameters for the micelles at the beginning of the drying process (Table S1); SasView model fit to the initial SAXS profile of the 20 wt % droplet (Figure S2); cross-polarized timelapse images and schematic locating the hexagonal phase at the droplet surface (Figure S3); volume change of a 20 wt % droplet during drying at 60 °C (Figure S4); side-by-side comparison showing droplet darkening during drying (Figure S5). (PDF)
