# Supplementary material for: Time Resolved Small-Angle X‑ray Scattering of Lyotropic Phase Evolution in Drying Levitated Aqueous Alpha Olefin Sulfonate Droplets
Source: Langmuir. 2026 Oct 26;42(44):32709–20. doi: 10.1021/acs.langmuir.6c04771 (PMC13757944; doi:10.1021/acs.langmuir.6c04771)
Supplement: Supplementary file 1 [file la6c04771_si_001.pdf]

Supporting Information

Time Resolved Small-Angle X-ray Scattering of Lyotropic Phase Evolution in Drying Levitated Aqueous Alpha Olefin Sulfonate Droplets

Robin Winder[1*], Sepideh Khodaparast[2], Gabriele Sumanskaite[1], Andrew Bayly[1], Ian McRobbie[3], Michael Rappolt[4], and David Harbottle[1*]

[1)] School of Chemical and Process Engineering, University of Leeds, Leeds LS29JT, UK
[2)] School of Mechanical Engineering, University of Leeds, Leeds LS29JT, UK
[3)] Innospec Ltd, Oil Sites Road, Ellesmere Port, Cheshire, CH65 4EY, UK
[4)] School of Food Science and Nutrition, University of Leeds, Leeds LS29JT, UK

*Corresponding author e-mail: D.Harbottle@leeds.ac.uk, cp18rw@leeds.ac.uk

Contents

Figure S1. Time-resolved SAXS contour plots of repeat droplet-drying experiments...........S2
Table S1. Fitting parameters for the micelles at the beginning of the drying process S2
Figure S2. SasView model fit to the initial SAXS profile of the 20 wt% droplet..................S3
Figure S3. Cross-polarised timelapse images and schematic locating the hexagonal phase at the droplet surface S3
Figure S4. Volume change of a 20 wt% droplet during drying at 60 °C...............................S4
Figure S5. Side-by-side comparison showing droplet darkening during drying S4

To assess the reproducibility of the drying experiments, repeat measurements were performed on separate droplets. A representative selection of these repeats is shown in Figure S1. In each

repeat, the droplet passed through the same sequence of lyotropic phases, with the characteristic peaks appearing at comparable q positions and at similar stages of drying. These results demonstrate good repeatability and confirm that the phase evolution described in the main text is representative of the system rather than specific to a single droplet.

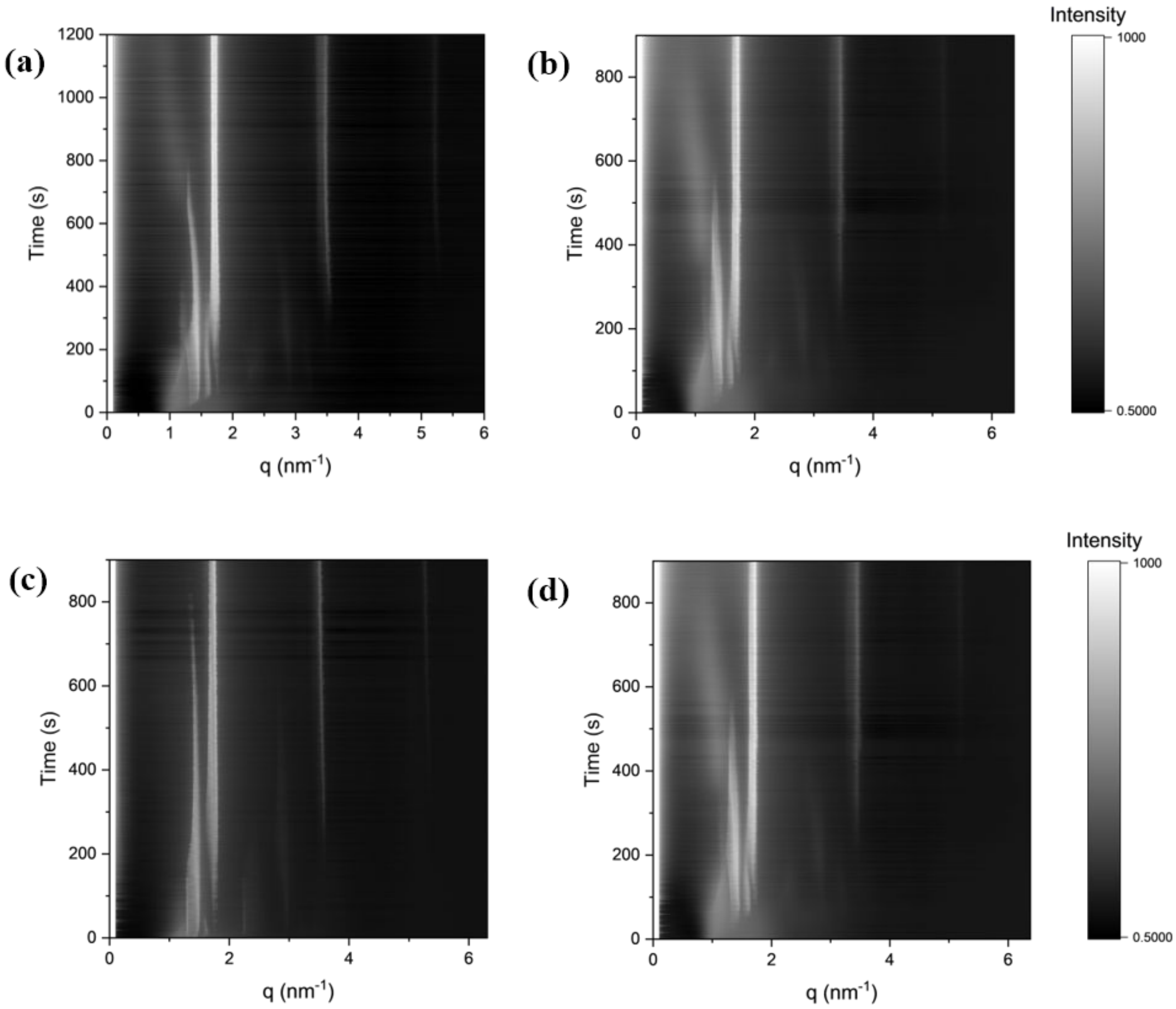

Figure S1. Time-resolved SAXS contour plots of droplet drying: (a, b) repeat experiments at 20 wt% and 60 °C; (c) 40 wt% at 60 °C; (d) 20 wt% at 40 °C.

Table S1. Table showing the fitting data for the micelles at the beginning of the drying process.

**Parameter**	**Value**
Core equatorial semi-axis	21.1 ± 1.6 Å
Core polar semi-axis	31.7 Å
Core axial ratio	1.5 ± 0.6
Shell thickness	5.4 ± 0.5 Å
Micelle equatorial semi-axis	26.5 Å
Micelle polar semi-axis	37.1 Å
Volume fraction	0.344 ± 0.013
Effective charge	24 ± 7 e

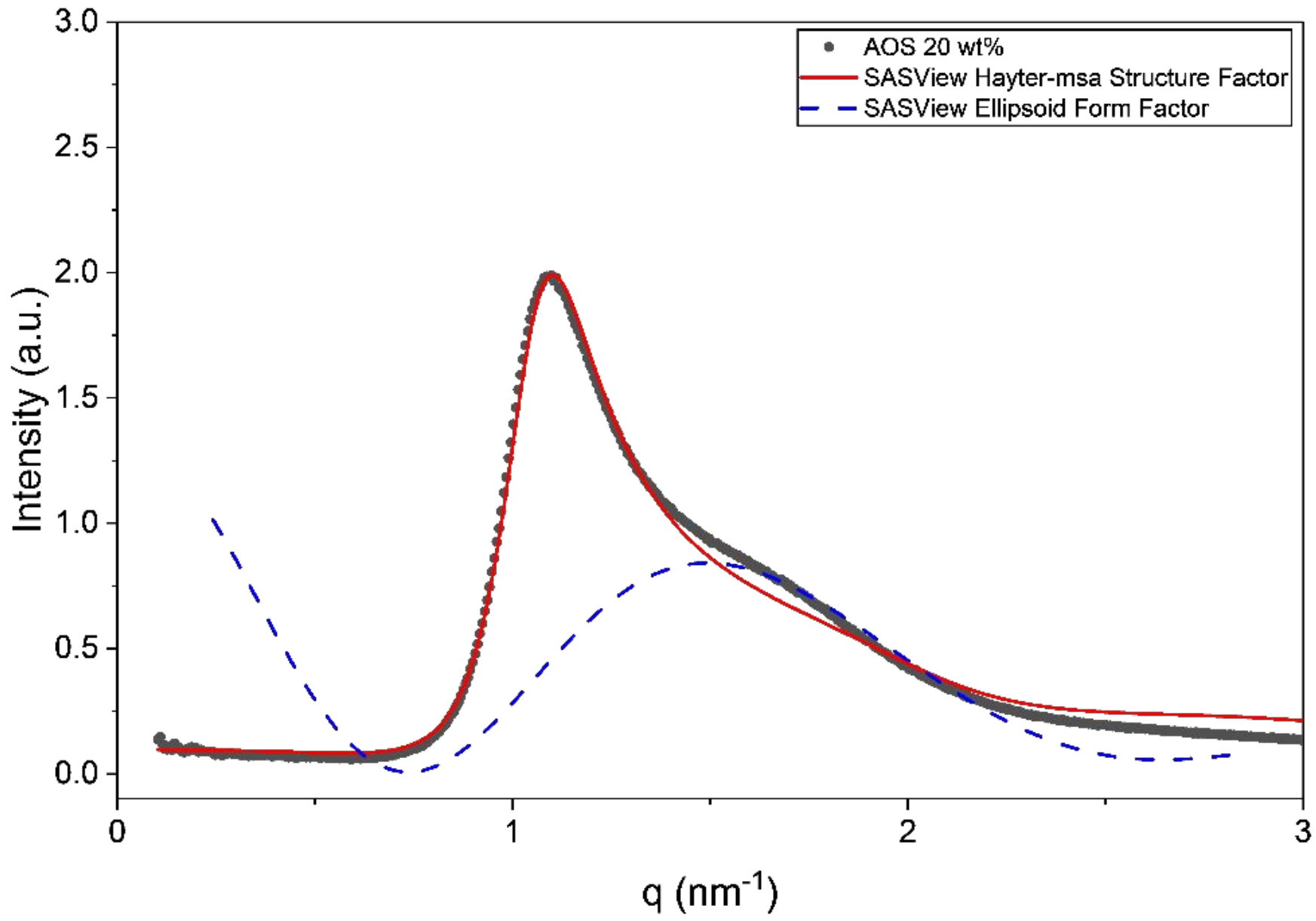

Figure S2. A graph showing the SASVIEW fitting in comparison with the initial starting condition of the 20 wt% sample with the addition of the ellipsoidal micelle without the structure factor.

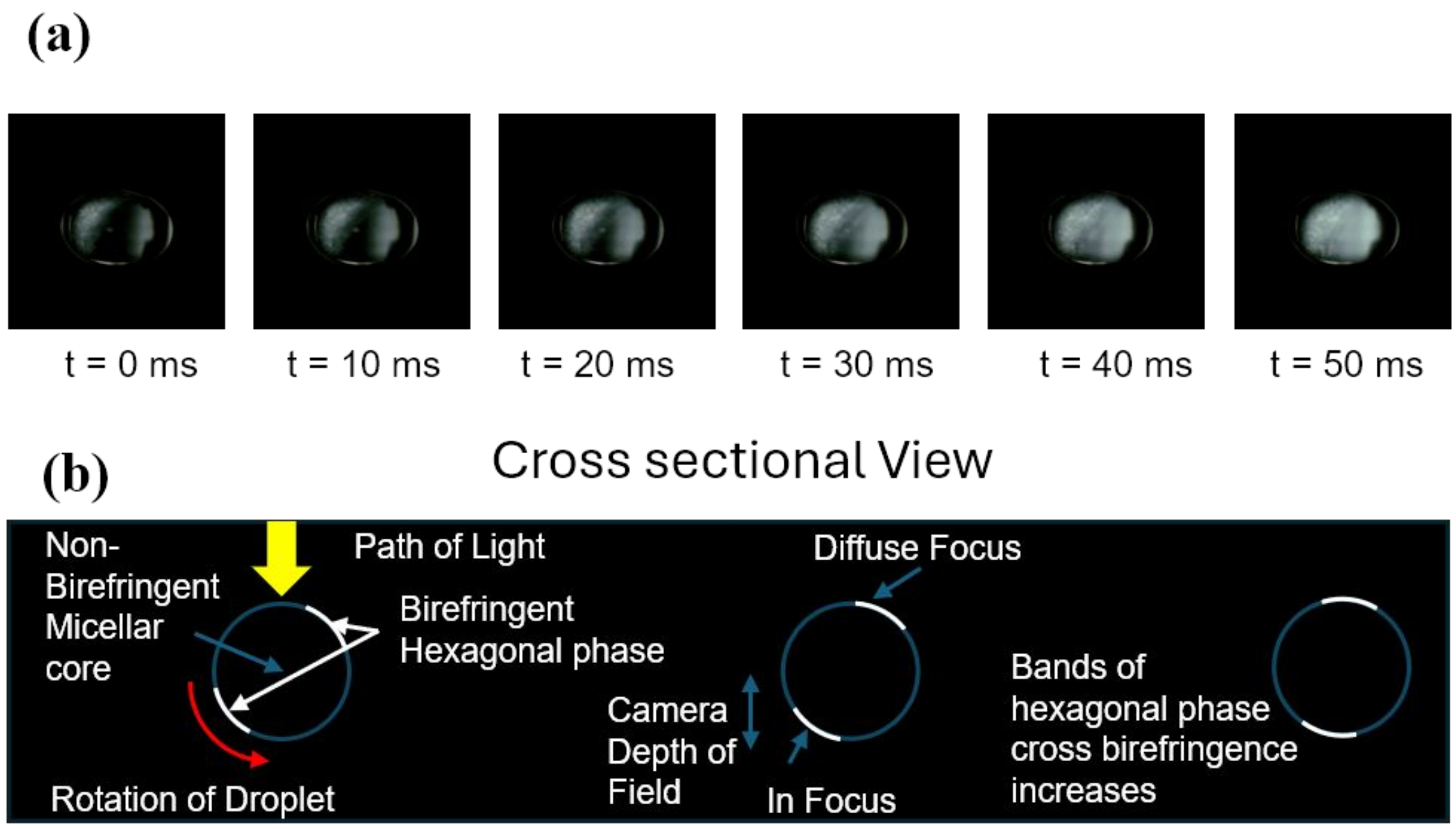

Figure S3. (a) A cross polarized timelapse images captured at 100 fps. Two distinct bands are initially present with an optically transparent gap between them. The nearest band retains focus and the rear band is diffuse. This is consistent with the depth of field of the camera system and places them at different distances along the optical axis (t = 0-20 ms). The gap narrows until the bands begin to cross each other (t = 30-50 ms). The combined retardation hence the transmitted intensity is observed to increase in comparison to the separated bands this would not happen if the hexagonal phase was located in the centre of the droplet confirming the location of the hexagonal phase to be at the surface of the droplet rather than the interior. (b) A schematic of the cross section of the droplet detailing the position of the hexagonal phases and the rotation of the droplet.

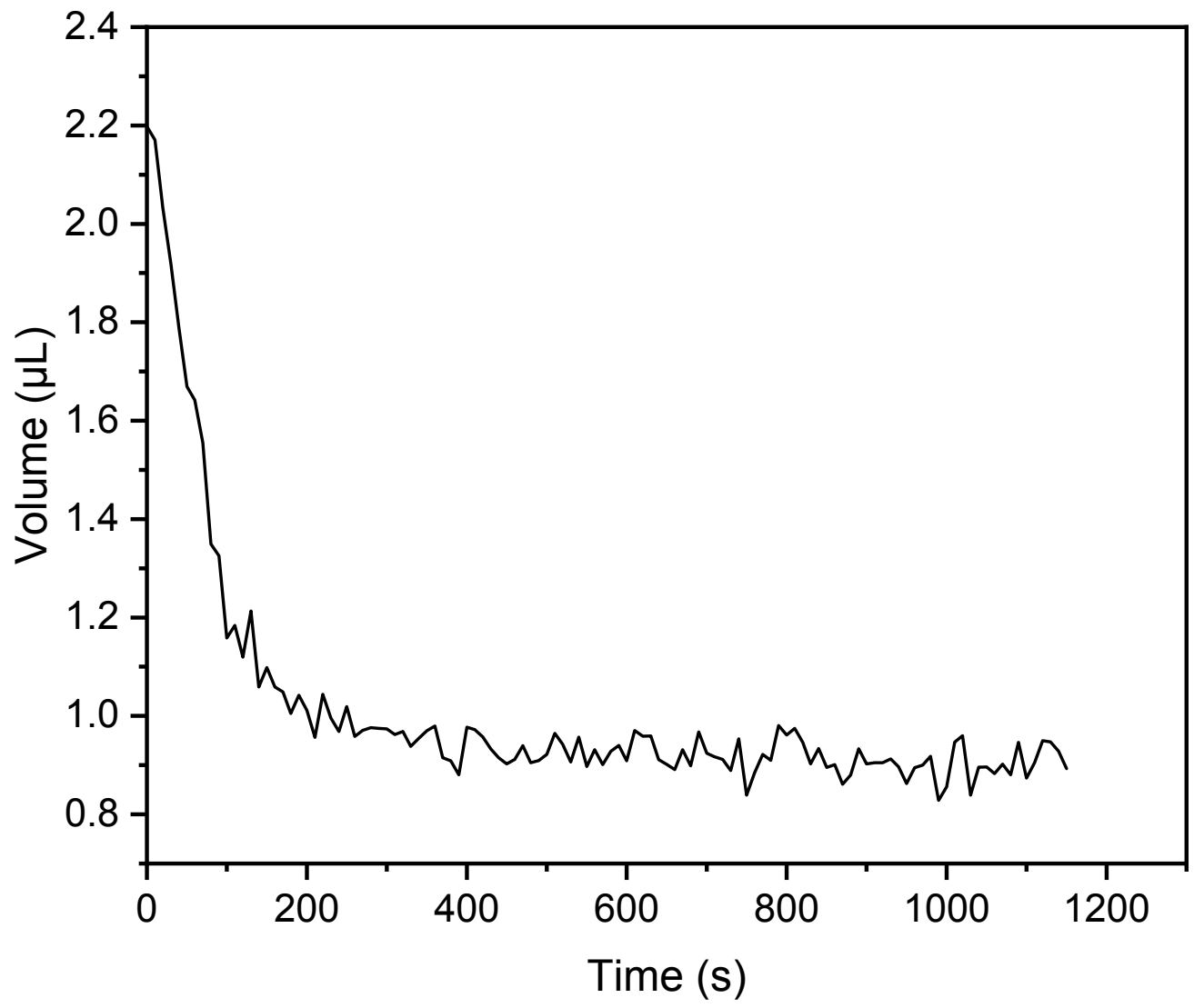

Figure S4. Volume change of a 20 wt% droplet during drying at 60 °C. The volume remained largely constant after 200 s of drying. The volume change was determined by filming the droplets and measuring the principal axes using a custom MATLAB script. The script then converted this to a volume measurement by treating each droplet as an oblate spheroid.

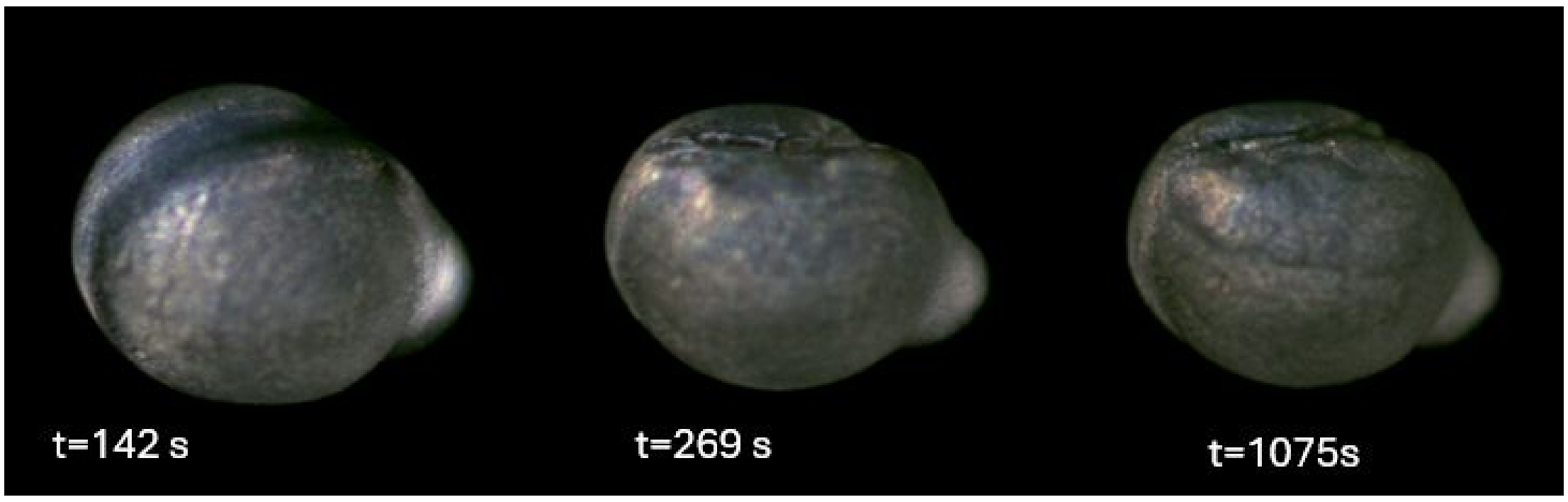

Figure S5. Side-by-side comparison of the droplets, showing that the droplet darkens over time.
